# Effects of direct oral anticoagulants on cardiac outcomes in atrial fibrillation: a systematic review and network meta-analysis

**DOI:** 10.1007/s11739-026-04480-1

**Published:** 2026-08-03

**Authors:** Danilo Menichelli, Daniele Malatesta, Arianna Pannunzio, Alessio Farcomeni, Francesco Violi, Pasquale Pignatelli, Daniele Pastori

**Affiliations:** 1 Department of General Surgery, Surgical Specialty and Anesthesiology, Sapienza University of Rome, Rome, Italy; 2 Postgraduate School of Internal Medicine, Department of Internal Medicine and Medical Specialties, Sapienza University of Rome, Rome, Italy; 3 Faculty of Economics, Tor Vergata University of Rome, 00133 Rome, Italy; 4 Sapienza University of Rome, Rome, Italy; 5 Department of Medical and Cardiovascular Sciences, “Sapienza” University of Rome, Viale del Policlinico 155, 00161 Rome, Italy; 6 IRCCS Neuromed, Località Camerelle, Pozzilli, Italy

**Keywords:** Direct oral anticoagulants, Myocardial infarction, Atrial fibrillation, Vitamin K antagonist, Elderly, Major cardiovascular events, Edoxaban, Apixaban, Rivaroxaban, Dabigatran

## Abstract

**Background:**

Direct oral anticoagulants (DOACs) reduce thromboembolism in atrial fibrillation (AF), but their effect on cardiac outcomes is less studied.

**Methods:**

Systematic review and network meta-analysis were performed on AF patients on DOACs/vitamin K antagonists (VKAs). Both observational studies and randomized clinical trials (RCTs) were included. Endpoints were myocardial infarction (MI) and major adverse cardiac events (MACE). Rankograms and SUCRA were performed. Sub-group analysis included age (</≥ 75 years) and length of follow-up (</≥ 12 months).

**Results:**

50 studies (3 RCTs, 4 post hoc analyses of RCTs and 43 observational studies) with 1,769,987 patients were included (59.9% on DOACs and 45.3% women).

The MI risk (46 studies with 1,554,704 patients) was lower in all DOACs compared to VKA (apixaban: hazard ratio [HR] 0.83, 95% credible interval [95%CI 0.72–0.96], edoxaban: HR 0.68, 95%CI 0.53–0.86, rivaroxaban: HR 0.84, 95%CI 0.74–0.95, dabigatran: HR 0.85, 95%CI 0.75–0.97). SUCRA showed edoxaban as first choice for preventing MI overall. In patients aged < 75 years, a lower MI risk was found for edoxaban (HR 0.60, 95%CI 0.41–0.85), while in those aged ≥ 75 years, no significant difference was found among anticoagulants. Heterogeneity was low in all analyses. Network meta-analysis showed no differences among DOACs.

Compared to VKA, apixaban (HR 0.77, 95%CI 0.63–0.97) was associated with lower MACE risk. Analysis of SUCRA showed apixaban as first choice for preventing MACE overall and in patients treated for < 12 months.

**Conclusion:**

DOACs were associated with a lower risk of MACE/MI in AF. The effect of DOACs on cardiovascular risk differs according to aging and follow-up length. However, our findings are based on indirect evidence, and further studies are needed.

**PROSPERO registration number:**

CRD42023407778.

**Supplementary Information:**

The online version contains supplementary material available at https://doi.org/10.1007/s11739-026-04480-1.

## Introduction

Direct oral anticoagulants (DOACs) showed at least similar efficacy compared to vitamin K antagonists (VKAs) for stroke prevention in atrial fibrillation (AF) with lower bleeding risks in both randomized trials and real-world studies [1, 2 ]. For this reason, they are recommended by current guidelines for thromboprophylaxis in non-valvular AF patients [1]. Despite optimal antithrombotic treatment, patients with AF still suffer an important rate of major adverse cardiovascular events (MACE) [3] including myocardial infarction (MI), heart failure with increased rate of hospital admission [4] and healthcare resources utilization [5].

Indeed, the incidence rate of MI is higher in patients with AF, with an incidence rate ratio of 3.12 compared to those without AF [6]. In the REGARDS study, AF patients had a twofold increased risk of MI compared to patients without AF, during a median follow-up of 4.5 years [7]. Notably, the risk of MACE seems to have a higher rate during first year from new-onset AF (4.7%, 95% CI, 3.9–5.6) declining then to 2.5% per year [8]. Furthermore, MI risk increases in the aging AF population, with a cumulative incidence of 3.3% in patients aged 67–69 years and of 4.4% in those aged 85–89 years [9].

Growing evidence shows a bidirectional correlation between AF and MI [10]. Indeed, both clinical conditions share risk factors including advanced age, hypertension, diabetes, previous stroke and cardiovascular events, metabolic syndrome, and dyslipidemia. Three main mechanisms can account for MI in AF patients: a high systemic inflammation level due to oxidative stress (1); a direct coronary thromboembolism from left atrial appendage (2); and tachyarrhythmia episodes that cause supply–demand mismatch (3) [10].

The treatment with VKAs seems to have some positive effects on the risk of MI [11]. However, the risk of MACE and MI seems to be greatly influenced by the quality of anticoagulation by VKAs [12]. As such, a good time in therapeutic range is associated with lower MACE risk [12]. However, a matter of concern was that the rate of MACE was still > 2%/year, suggesting that VKA may not be enough to lower the risk of MI in AF [10].

Data regarding the effectiveness of DOACs compared to VKA for the prevention of cardiovascular complications are conflicting [13, 14 ]; so far, there is no randomized clinical trial comparing DOACs among each other. For these reasons, the aim of this network meta-analysis is to evaluate the efficacy of DOACs to prevent MACE and MI compared with VKA in AF patients.

## Methods

### Search strategy and study selection process

We searched MEDLINE (PubMed) from initiation up to 31 st December 2025 to retrieve eligible records. The search strategy included “DOAC”, “NOAC”, “rivaroxaban”, “edoxaban”, “dabigatran”, “apixaban”, “myocardial infarction”, “AMI”, “heart attack”, “major adverse cardiac events” both as MeSH and free terms, combined with Boolean operators with no filters applied. The complete search strategy is outlined in Supplement S1. We included records written in English, reporting the results of observational studies and randomized clinical trials (RCTs) in the systematic review comparing different anticoagulant treatments (DOAC or VKA). We excluded review articles, commentaries, congress abstracts, single-arm studies, cross-sectional and cross-over articles, studies using antiplatelet or placebo as comparator. Two study authors (D.M. and A.P.) independently screened the retrieved citations by title and abstract. Potentially eligible articles were read in full text for final inclusion decision, and disagreements were solved by collegial discussion with a third independent author (D.M.).

This systematic review and network meta-analysis is registered on PROSPERO with the number: CRD42023407778. Compared to the original study design, we have added a second endpoint including major adverse cardiac events (MACE), and we decided to perform a network meta-analysis with prespecified sub-group analyses according to age and median follow-up.

We followed the PRISMA International Guidelines for the realization of the study [15]. Accordingly, the PRISMA checklist is reported in Supplementary Table 1.

### Participants/population

We included only studies that enrolled patients with non-valvular AF on oral anticoagulant therapy (VKA or DOACs).

### Interventions/comparator

To investigate the risk of MI and MACE in AF patients taking either DOACs or VKA, MACE did not include ischemic stroke/TIA.

### Assessment and definition of outcomes

Time-to-event efficacy outcomes included MI and MACE, during the follow-up period in patients taking oral anticoagulant therapy for AF alone or undergoing percutaneous coronary intervention (PCI).

We performed sub-group analyses according to age (above and under 75 years) and follow-up (median above and under 12 months)*.*

### Data extraction

Two authors (D.M. and A.P.) independently extracted data from included studies into an Excel spreadsheet. We collected the following information: first author, year of publication, clinical setting, total cohort, study design, the number of patients included and the number of patients taking DOACs and VKA, age, sex, follow-up duration, CHA_2_DS_2_-VASC score, HAS-BLED score, concomitant comorbidities such as proportion of patients with hypertension, diabetes, coronary heart disease (CHD), heart failure (HF), previous stroke or TIA, dyslipidemia, smoking habit, previous MI, patients in therapy with single antiplatelet therapy (SAPT) or dual antiplatelet therapy (DAPT) and included systematic review outcomes. Discrepancies were solved by collegial discussion.

### Risk of bias assessment and publication bias

Two authors (D.M. and A.P.) independently assessed the risk of bias (RoB) using the Cochrane Robins-I tool for observational studies [16], which evaluates the following domains: bias due to confounding, bias due to selection of participants, bias in classification of interventions, bias due to deviations from intended interventions, bias due to missing data, bias in measurement of outcomes, bias in selection of the reported results [16]. Two authors (D.M. and A. P.) independently assessed RoB using the Cochrane ROB2 tool [17] for RCTs, which evaluates the following domains: bias arising from the randomization process, bias due to deviations from intended interventions, bias due to missing outcome data, bias in measurement of the outcome, bias in selection of the reported result. Robins *I* and ROB2 figures were created with the robvis online tool [18].

### Statistical analysis

If at least two included studies had available data for assessing the primary endpoint, we performed a random effect Bayesian network meta-analysis to estimate the pooled hazard ratio (HR) with 95% credibility intervals (95% CI). RCTs and observational studies were analyzed separately. Posterior distributions were computed using Markov Chain Monte Carlo samplers, each based on 500,000 iterations: with a burn-in of 100,000 iterations. For each endpoint, we ran two parallel chains to check for convergence of the MCMC sampler. Heterogeneity was assessed with I^2^ statistics. As sensitivity analysis, a forest plot was finally used to visually assess that removal of each study in no case led to a leave-one-out pooled HR outside the 95% credible interval.

For each outcome, a network diagram was utilized to illustrate the amount of evidence available (Supplementary Fig. 1 for MACE and Supplementary Fig. 2 for MI**)**. The size of the nodes is proportional to the total number of patients included to the corresponding treatment across all studies, and the width of the lines is proportional to the total number of studies evaluating the corresponding treatment comparison.

For each outcome of interest, we retrieved from each study the HR derived from the multivariable adjusted Cox proportional hazard regression analysis or from propensity score matching. Summary of pooled results is expressed as hazard ratios (HRs) with 95% credible intervals.

When HRs were not reported, we estimated them from summary statistics with the methods described by Tierney [19]. Potential publication bias was evaluated through funnel plots and Egger’s linear regression test. Results are shown with rankograms. We analyzed separately patients with AF and patients with AF undergoing PCI. Sub-group analysis according to age (< 75 years and ≥ 75 years) and time of follow-up (< 12 months and ≥ 12 months) was performed. Additionally, Bayesian network meta-regression analyses were performed for use of antiplatelet and previous cardiovascular disease to assess for their possible role in affecting the efficacy of oral anticoagulant therapy in preventing MI in AF patients. R software version 4.3.3 was used throughout.

### Ethical approval and informed consent

Ethical approval was not required due to study type (systematic review and network meta-analysis). Patients were not involved in the design and the development of this study.

## Results

### Search results and study characteristics

We identified 1304 references from literature. After the review process (Supplementary Fig. 3), 50 studies met the inclusion criteria for quantitative synthesis (38 retrospective, 5 prospective, 4 post hoc analysis of RCTs and 3 RCTs) and were included in the network meta-analysis as shown in the PRISMA flowchart diagram.

The overall population consisted of 1,769,987 participants (Table 1), of whom 1,059,577 (59.9%) were treated with DOACs, 231,043 were on dabigatran, 148,138 on edoxaban, 260,275 on apixaban, and 416,707 on rivaroxaban.

**Table 1:** Clinical characteristics studies included in the meta-analysis

Year	First author/cohort	Design	Total cohort	DOAC (%)	Female (%)	Age (mean)	CHA_2_DS_2_-VASc	HAS BLED	Hypertension (%)	Diabetes (%)	Previous CVE (%)	HF (%)	Previous stroke/TIA (%)	Follow-up (months)	SAPT (%)	DAPT (%)	Endpoint
*RCT—post hoc RCT*																	
2012	Hohnloser (RE-LY) [32]	PostH	18,113	66.8	36.4	71.5	–	–	78.9	23.3	16.6	–	20.0	14.0	45.1	3.6	MI
2013	ENGAGE-AF TIMI 48[33]	PostH	21,105	66.7	38.1	72.0	–	–	93.6	36.1	–	57.4	28.3	34.0	29.6	0.0	MACE/MI
2013	Bahit (ARISTOTLE) [34]	PostH	18,184	50.2	35.2	70.0	–	–	87.5	25.0	36.5	–	19.4	21.6	32.8	0.0	MI
2016	Gibson (PIONER-AF) [21]	RCT	2124	66.6	25.5	70.1	3.8	3.0	74.0	29.0	23.0	24.6	–	12.0	33.4	66.6	MACE/MI
2018	Barnett (ROCKET AF) [49]	PostH	14,236	49.9	40.0	73.0	3.5	–	90.5	39.9	17.3		54.7	24.0	36.5*	–	MI
2019	Oldgren (RE-DUAL PCI trial) [57]	RCT	2725	64.0	24.0	70.8	3.6	2.7	–	36.0	25.7	–	8.3	14.0	64.0	36.0	MI
2020	Vranckx (ENTRUST-AF PCI trial [23]	RCT	1506	49.9	25.5	69.7	4.0	3.0	90.5	34.0	24.0	55.0	12.5	12.0	100.0	–	MI
*Observational studies*																	
2014	Larsen [35]	R	13,914	36.0	42.5	70.3	–	–	19.6	12.0	9.5	–	17.3	10.5	39.6	0.0	MI
2015	Graham [36]	R	134,414	50.0	51.5	–	–	–	87.0	33.5	18.0	18.0	23.0	–	17.0	–	MI
2015	Lauffenburger [37]	R	64,935	32.5	39.9	69.9	2.7	–	71.8	30.1	32.3	25.1	9.5	11.9	13.0	–	MI
2015	Seeger–MARKETSCAN [38]	R	31,058	50.0	37.8	68.5	3.1	2.2	96.4	20.6	30.0	17.8	12.8	4.5	13.1	–	MI
2015	Seeger–Clinformatics [38]	R	7320	50.0	30.2	63.2	2.8	2.2	96.4	28.4	34.4	18.1	16.0	4.5	10.2	–	MI
2015	Chan [39]	R	19,853	50.1	42.0	75.5	4.1	2.6	87.0	41.0	3.0	16.0	38.0	8.4	46.5	–	MI
2015	Villines [40]	R	25,586	50.0	41.2	73.9	3.9	3.4	96.1	14.7	19.6	12.6	5.2	8.6	–	–	MI
2017	Coleman [41]	R	1670	50.0	48.6	75.1	3.6	–	65.6	22.2	–	14.5	–	–	28.5	–	MI
2017	Go [42]	R	50,578	50.0	35.9	68.4	–	–	81.6	29.8	7.1	38.3	–	7.5	13.3*	–	MI
2017	Lai [43]	R	9200	100.0	45.3	75.2	–	3.3	49.6	20.3	4.7	26.2	19.3	10.8	–	–	MI
2017	Norby [44]	R	33,914	100.0	34.2	67.2	2.6	–	60.6	23.5	5.4	19.4	12.2	12.0	–	–	MI
2017	Norby [44]	R	77,991	41.7	40.0	70.4	3.1	–	64.1	26.3	7.7	24.8	16.5	12.0	–	–	MI
2017	Shantha [45]	R	33,111	66.7	100.0	76.8	4.8	1.6	86.6	31.4	6.9	23.4	12.6	14.0	5.4*	–	MI
2017	Shantha [45]	R	22,827	66.7	0.0	74.9	3.8	1.7	84.0	35.8	10.7	24.8	11.4	14.0	7.2*	–	MI
2018	Yu [46]	R	11,712	50.0	42.3	70.5	4.5	–	94.5	32.5	13.3	65.0	38.8	5.0	22.2	–	MI
2018	Lai [47]	R	4722	68.3	52.0	88.6	3.8	–	51.3	15.9	1.4	27.8	14.5	6.6	54.9	–	MI
2018	Lee [48]	R	31,739	72.0	46.3	73.8	2.3	–	61.0	11.6	7.9	15.7	16.0	36.0	35.0	4.0	MI
2018	Själander [50]	R	64,382	42.2	41.4	74.0	3.1	–	61.0	17.4	17.4	12.8	22.2	11.5	–	–	MI
2018	Briasoulis [51]	R	85,596	32.5	53.0	77.0	4.4	1.7	85.0	37.0	9.7	25.8	–	12.0	5.0	–	MI
2018	Hsin-Fu Lee [52]	R	42,000	61.9	48.0	78.0	4.0	3.0	86.0	39.0	13.0	14.0	22.0	15.6	–	–	MI
2018	Mueller [53]	R	14,577	100.0	45.3	74.0	2.9	2.0	36.7	15.2		14.1	14.3	9.0	42.0	–	MI
2019	Pratt [54]	R	4836	71.8	48.0	81.9	2.2	–	–	–	–	–	–	12.0	38.7	2.8	MI
2019	Amin [55]	R	198,171	58.9	50.1	78.2	4.6	3.3	88.4	37.6	13.2	32.2	22.0	6.0	20.9	–	MACE
2019	Russo [20]	P	899	43.3	43.0	71.2	3.8	2.7	54.5	16.5	10.5	24.5	34.0	6.0	100.0	–	MACE
2019	Baker [56]	R	24,646	43.3	36.5	70.0	4.0	–	86.5	100.0	10.2	33.2	9.0	16.8	16.1*	–	MACE/ MI
2020	Chan [58]	R	26,779	78.3	46.3	74.5	4.4	3.1	80.0	100.0	14.0	13.2	21.3	60.0	22.3	–	MACE/ MI
2020	Coleman [59]	R	8303	39.2	36.0	74.0	3.0	2.0	86.0	46.3	10.0	40.9	11.0	16.8	28.1	–	MACE/ MI
2021	Ingason [60]	R	4670	100.0	40.4	72.0	2.6	–	67.6	4.1	7.8	7.4	7.7	72.0	24.3	0.0	MI
2021	Briasoulis [61]	R	28,011	52.1	10.5	67.1	–	–	85.5	28.4	4.7	–	–	20.5	–	–	MI
2021	Lopes [62]	R	155,038	57.2	45.9	78.7	4.3	3.6	94.3	44.0	17.4	–	–	11.0	–	–	MI
2021	Elis [63]	P	252	38.5	46.8	78.7	4.9	2.5	76.2	49.5	43.7	58.3	15.5	12.0	–	–	MI
2021	Crocetti [64]	R	8543	87.1	48.7	77.0	4.4	–	–	–	–	–	–	14.0	4.2*	–	MI
2021	Lopes [65]	R	94,435	52.7	45.3	78.7	4.3	3.6	94.3	44.0	17.5	40.5	26.0	12.0	24.7*	–	MI
2022	Liu [66]	R	1246	63.2	41.5	71.0	–	–	71.9	19.6	–	29.4	18.3	61.0	–	–	MI
2022	GLORIA-AF [67]	P	12,359	100.0	45.2	71.7	3.2	1.3	76.2	23.2	14.4	17.7	10.7	36.0	17.8	0.0	MI
2022	Boivin-Proulx [68]	R	1656	24.3	55.8	72.6	2.6	3.4	87.5	60.1	12.2	–	–	12.0	6.2	–	MI
2022	Boivin-Proulx [68]	R	1792	30.1	56.1	74.2	2.7	3.4	89.5	61.5	13.5	–	–	12.0	5.4	–	MI
2022	Yu [69]	R	319	55.8	47.0	80.0	5.1	2.3	69.6	26.3	31.0	–	30.1	36.0	26.0	12.0	MI
2022	Jaksa [70]	R	5655	100.0	36.9	77.0	3.4	–	73.7	27.1	–	12.1	–	26.7	33.9	0.0	MI
2023	Talmor-Barkan [71]	R	56,553	100.0	51.3	77.3	4.2	3.0	89.4	47.0	17.3	31.2	8.4	72.0	29.0	0.0	MI
2023	Grymonprez [72]	R	254,478	75.8	47.4	75.0	3.5	2.4	64.7	32.5	15.7	15.7	–	72.0	45.0	0.0	MI
2023	Pereira [73]	P	53	49.0	26.0	66.0	4.0	1.0	93.0	55.0	100.0	–	11.0	1.0	100.0	0.0	MACE
2024	Burnham [22]	R	333	100.0	57.0	71.0	4.0	2.0	90.0	39.0	–	18.0	13.0	–	–	–	MACE/MI
2024	Kreutz [74]	P	1455	48.0	44.0	78.0	–	–	80.0	40.0	13.0	22.0	8.0	24.0	–	–	MI
2024	Liu [75]	R	1301	100.0	41.0	71.0	–	–	72.0	20.0	–	30.0	23.0	24.0	–	–	MI
2025	Dari [76]	R	16,160	100.0	44.0	79.0	–	–	78.0	54.0	19.0	23.0	21.0	24.0	59.0	–	MACE
2025	Fang [77]	R	2952	50.0	56.0	82.0	5.0	3.0	78.0	31.0	5.0	32.0	44.0	–	–	–	MI

*ACS* acute coronary syndrome, *CAD* coronary artery disease, *CCD* chronic cardiovascular disease, *CKD* chronic kidney disease, *CLI* critical limb ischemia, *HF* heart failure, *MACE* major adverse cardiac events, MI myocardial infarction, *NVAF* non-valvular atrial fibrillation, *P* prospective, *PAD* peripheral artery disease, *PCI* percutaneous coronary intervention, *PostH* post hoc of randomized clinical trial, *R* retrospective, *RCT* randomized clinical trial, *SAPT* single antiplatelet therapy, *DAPT* dual antiplatelet therapy

*Reported as antiplatelets

45.3% were women with an age ranging between 63.2 and 88.6 years.

Among the cardiovascular risk factors: 89.2% of the population had hypertension, 39.3% diabetes, 13.3% previous CVE, and 5.9% HF. 3.08% of patients were assuming SAPT as reported in Table 1.

The number of patients in each anticoagulant group is reported in Supplementary Table 2.

### Risk of bias assessment

The risk of bias of 5 RCT is reported in Supplementary Fig. 4 Panel A and B (traffic light plot and summary plot with ROB2). Two studies were considered at low risk of bias due to randomization process, to deviations from intended interventions, due to missing outcome data, in measurement of the outcome, in selection of the reported results. Three studies had some concerns in the domain of missing outcome data. Overall, 100% of studies included had a low risk of bias for domains 1, 2, 4, and 5. The risk of bias of 45 observational studies included in meta-analysis is reported in Supplementary Fig. 4 Panel C and D. Three studies were considered at low risk of bias due to confounding, bias in classification of interventions, due to deviations from intended interventions, due to missing data, in measurement of outcome and in selection of reported results. Thirty-six studies had moderate risk of bias due to confounding, bias in classification of interventions, due to deviations from intended interventions, due to missing data, and in selection of the reported results. Six studies had a high risk of bias due to bias due to confounding, bias due to deviations from intended interventions, bias due to missing data, bias in measurement of outcomes or bias in selection of the reported results. Overall, at least 75% of studies included had a low risk of bias for domains 3, 4, 5, 6, and 7 of ROBINS-I tool.

Confounders evaluated and adjusted for in each study included in the meta-analysis are reported in Supplementary Table 3.

### MACE

**Fig. 1:**
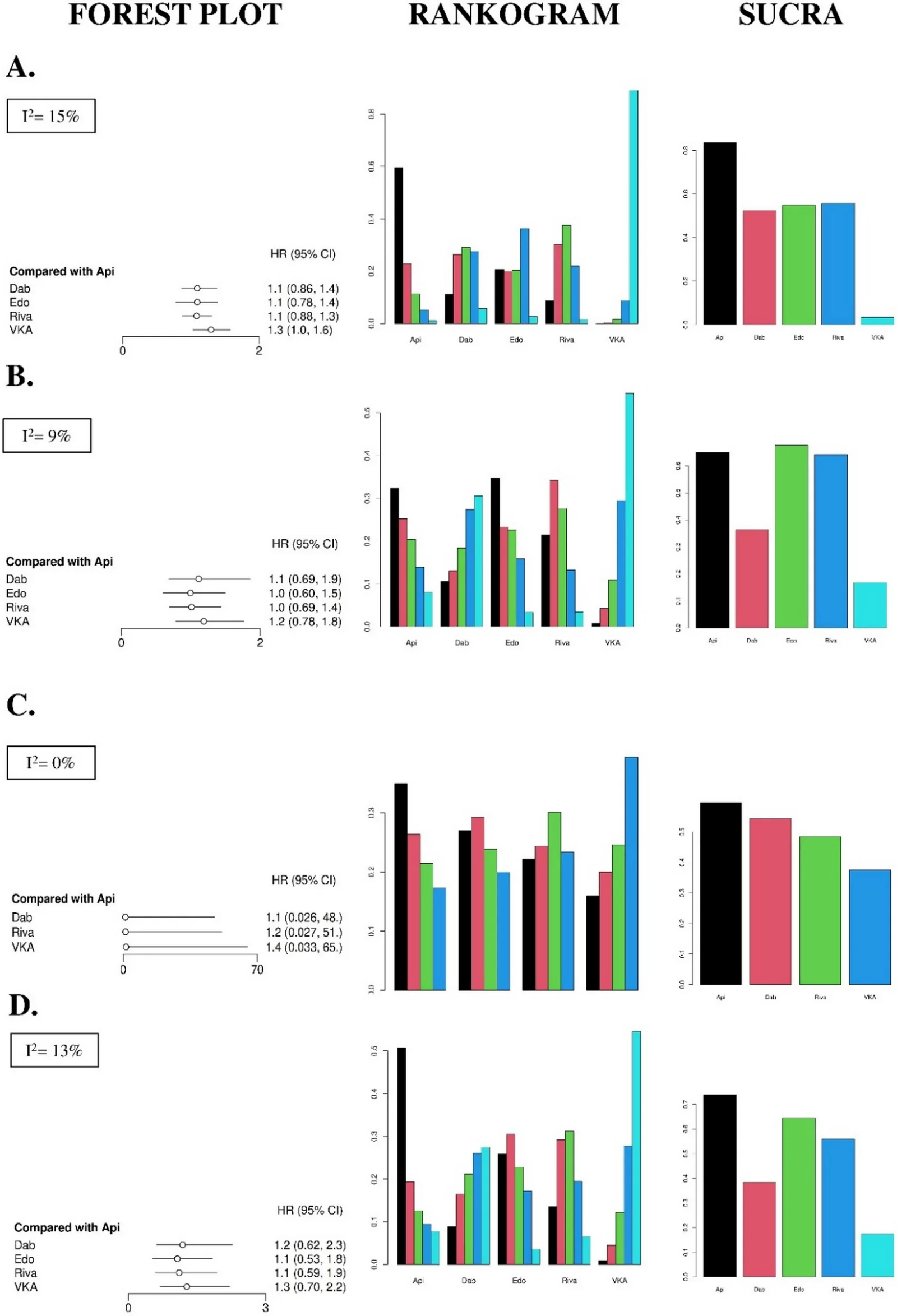
Major adverse cardiac events (MACE) in observational studies included in the meta-analysis. **A** Overall analysis in patients with atrial fibrillation (AF) alone (8 observational studies). **B** Patients with AF and a median follow-up ≥ 12 months (5 observational studies). **C** Patients with AF and a median follow-up < 12 months (2 observational studies). **D** Patients with AF and a median age < 75 years (6 observational studies). *Api* apixaban, *CI* credible interval, *Dab* dabigatran, *Edo* edoxaban, *HR* hazard ratio, *Riva* rivaroxaban, *SUCRA* surface under the cumulative ranking curve, *VKA* vitamin K antagonist

MACE endpoint was not available in RCTs. MACE endpoint was reported in 8 observational studies (Table 1), including 295,550 patients with AF alone, of whom 182,275 (61.7%) were on DOACs. Compared to apixaban, the pooled analysis showed no difference in MACE risk in patients treated with rivaroxaban, dabigatran, or edoxaban, and VKA (Fig. 1, Panel A). Heterogeneity was low (*I*^2^ 15%).

MACE in patients with AF undergoing PCI were reported in 2 studies (1,765 patients, of whom 884 were on DOACs) included in the systematic review [20, 21 ]. However, they could not be meta-analyzed due to different study designs (observational and RCT).

We performed sub-group analysis according to median follow-up and elderly. For 5 and 2 observational studies reporting data on MACE in AF patients with a median follow-up < and ≥ 12 months, respectively (Fig. 1, Panel B, C), the sub-analysis confirmed the results of the main one. One study [22] was excluded from this analysis due to lack of information about length of follow-up.

For 6 observational studies reporting on MACE in AF patients < 75 years (Fig. 1, Panel D), the analysis confirmed the main results. All sub-group analyses had low heterogeneity ranging from 0 to 13% (Fig. 1, Panel B–D). A sub-group analysis including studies with a median age ≥ 75 years was not performed due to lack of sufficient studies to analyze. Funnel plots (Supplementary Fig. 5) suggested potential publication bias on MACE endpoint.

Analysis of SUCRA (Fig. 1, Panel A–D) showed apixaban as first choice for MACE prevention in the main analysis and in patients treated for < 12 months or younger than 75 years, while dabigatran seems to be the first choice in patients treated ≥ 12 months.

### Myocardial infarction

**Fig. 2:**
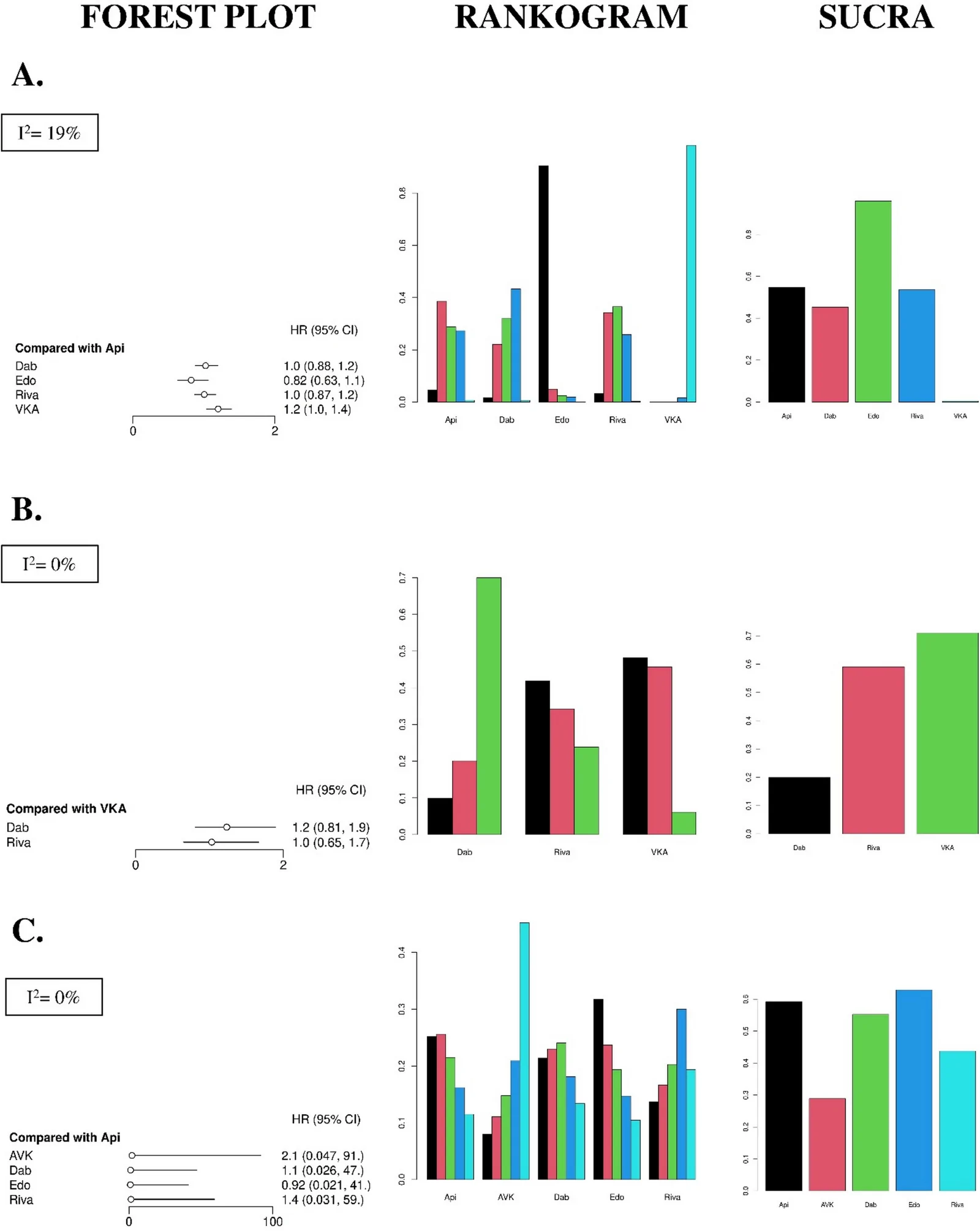
Myocardial infarction (MI) risk. **A** In patients with atrial fibrillation (AF) alone (46 observational studies). **B** In 3 clinical trials including patients with AF and concomitant percutaneous coronary intervention (PCI). **C** In 2 observational studies including patients with AF and PCI. *Api* apixaban, *CI* credible interval, *Dab* dabigatran, *Edo* edoxaban, *HR* hazard ratio, *Riva* rivaroxaban, *SUCRA* surface under the cumulative ranking curve, *VKA* vitamin K antagonist

The endpoint MI was reported in 46 observational studies including 1,554,704 patients (Table 1). Compared to apixaban, the pooled analysis showed no difference among DOACs (Fig. 2, Panel A) and a lower risk of MI in apixaban, compared to VKA (Fig. 2, Panel A) in patients with AF alone. Analysis of SUCRA (Fig. 2) showed edoxaban as first choice for MI prevention. Heterogeneity was low (I^2^ 19.0%).

**Fig. 3:**
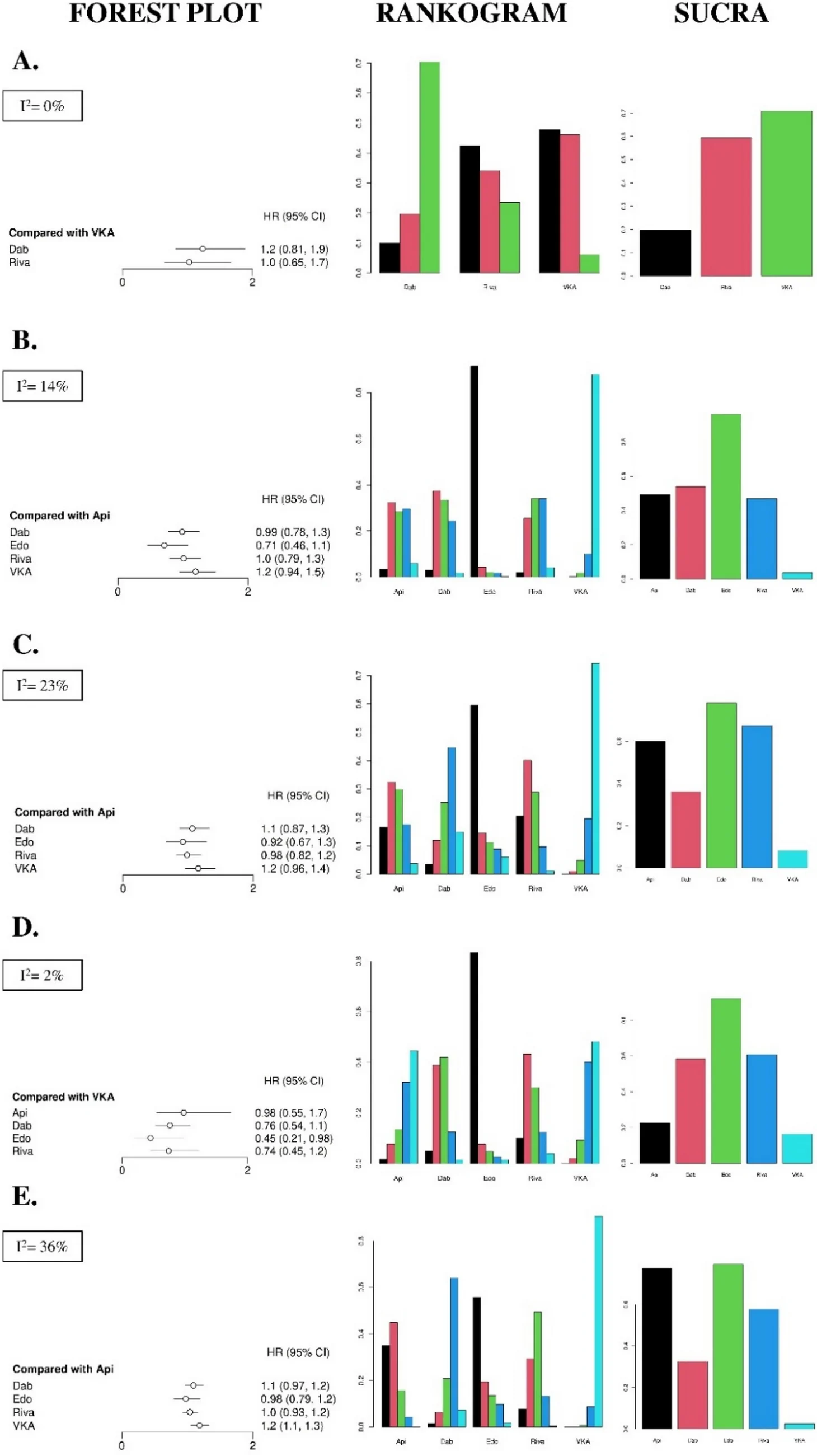
Sub-group analysis on myocardial infarction (MI) in: **A** patients < 75 years with atrial fibrillation (AF) and concomitant percutaneous coronary intervention (PCI) (3 RCTs), **B** Patients < 75 years with AF alone (26 observational studies), **C** patients ≥ 75 years with AF alone (17 observational studies), **D** patients with AF alone and a median follow-up < 12 months (11 observational studies), **E** patients with AF alone and a median follow-up ≥ 12 months (27 observational studies). *Api* apixaban, *CI* credible interval, *Dab* dabigatran, *Edo* edoxaban, *HR* hazard ratio, *Riva* rivaroxaban, *SUCRA* surface under the cumulative ranking curve, *VKA* vitamin K antagonist

In AF patients undergoing PCI, the three RCTs did not show significant difference among dabigatran, rivaroxaban, and VKA (Fig. 2, Panel B), and similar results were obtained between each DOAC and VKA in the analysis of two observational studies investigating AF patients undergoing PCI (Fig. 2, Panel C). Heterogeneity was low in both analysis (I^2^ 0.0% and 0.0%, respectively). No significant difference was observed in the sub-group analysis including three RCTs according to patients < 75 years in this cohort (Fig. 3, Panel A, *I*^2^ 0.0%). Sub-analyses including patients with median age ≥ 75 years and according to median follow-up were not performed in patients with AF undergoing PCI due to lack of studies that could be included.

We also performed sub-group analyses according to the length of follow-up and age in patients with AF alone.

For patients aged < 75 years, 26 observational studies were included. Compared with apixaban, the pooled analysis showed no significant difference among DOACs (Fig. 3, Panel B) with edoxaban as first choice at SUCRA. Heterogeneity was low (*I*^2^ 14%).

For patients ≥ 75 years of age, the analysis included 17 observational studies. Compared with apixaban, the pooled analysis showed no significant difference among DOACs (Fig. 3, Panel C) with edoxaban and rivaroxaban as first and second choice, respectively, at SUCRA analysis. Heterogeneity was low (*I*^2^ 23%).

For studies reporting on MI with a median follow-up < 12 months, the analysis included 11 observational studies. Compared with VKA, the pooled analyses showed a lower risk in the edoxaban group (HR 0.45, 95%CI 0.21–0.98), while no difference was observed among other DOACs and VKA (Fig. 3, Panel D). Heterogeneity was low (*I*^2^ 2%).

For studies reporting on MI with a median follow-up ≥ 12 months, the analysis included 27 observational studies. Compared to apixaban, the pooled analysis showed no difference among DOACs with edoxaban and apixaban as first and second choice at SUCRA analysis (Fig. 3, Panel E). Heterogeneity was low (*I*^2^ 36%). Heterogeneity for each endpoint was reported as *I*^2^ in Figs. 1, 2 and 3.

### Network meta-analysis

All DOACs were associated with a lower risk of MI compared to VKA, and results were consistent across various DOACs. However, apixaban was also associated with a lower MACE risk compared to VKA (Table 2, Panel A and B) in patients with AF alone. However, in the network meta-analysis, there were no differences in the relative effect among DOACs (Table 2 Panel A also reported HR and relative 95% CI of all comparisons among DOACs in the observational studies group and Panel B in the RCTs group). No difference was observed among anticoagulant treatment in patients with AF undergoing PCI.

**Table 2:** Network meta-analysis comparing DOACs and VKA in patients with atrial fibrillation alone for each endpoint according to: A. Observational studies, B. randomized clinical trial (RCT)

A. Observational					
Outcomes HR (95%CI)	Edoxaban				
MACE overall	1.09 (0.78–1.39)	Apixaban			
MI overall	0.82 (0.63–1.06)				
MACE ≥ 12 months	1.00 (0.60–1.50)				
MACE < 12 months	–				
MACE < 75 years	1.07 (0.53–1.83)				
MI ≥ 12 months	0.98 (0.78–1.19)				
MI < 12 months	0.46 (0.18–1.21)				
MI ≥ 75 years	0.92 (0.67–1.28)				
MI < 75 years	0.71 (0.46–1.08)				
MACE overall	1.00 (0.71–1.28)	0.92 (0.72–1.16)	Dabigatran		
MI overall	0.80 (0.62–1.03)	0.98 (0.84–1.14)			
MACE ≥ 12 months	0.89 (0.51–1.39)	0.9 (0.54–1.46)			
MACE < 12 months	–	0.92 (0.02–38.74)			
MACE < 75 years	0.91 (0.45–1.51)	0.84 (0.44–1.62)			
MI ≥ 12 months	0.89 (0.73–1.08)	0.91 (0.81–1.03)			
MI < 12 months	0.59 (0.25–1.38)	1.29 (0.75–2.21)			
MI ≥ 75 years	0.86 (0.62–1.19)	0.94 (0.75–1.15)			
MI < 75 years	0.72 (0.47–1.07)	1.01 (0.8–1.29)			
MACE overall	1.01 (0.75–1.26)	0.92 (0.77–1.14)	1.01 (0.82–1.27)	Rivaroxaban	
MI overall	0.82 (0.63–1.05)	1.00 (0.86–1.15)	1.02 (0.89–1.17)		
MACE ≥ 12 months	0.99 (0.64–1.4)	0.99 (0.70–1.44)	1.10 (0.71–1.79)		
MACE < 12 months	–	0.84 (0.02–36.41)	0.91 (0.02–38.31)		
MACE < 75 years	0.97 (0.57–1.48)	0.90 (0.52–1.70)	1.06 (0.62–1.99)		
MI ≥ 12 months	0.94 (0.77–1.13)	0.96 (0.86–1.07)	1.05 (0.95–1.17)		
MI < 12 months	0.61 (0.24–1.54)	1.33 (0.75–2.35)	1.03 (0.66–1.63)		
MI ≥ 75 years	0.93 (0.68–1.29)	1.02 (0.83–1.22)	1.08 (0.91–1.29)		
MI < 75 years	0.71 (0.46–1.05)	0.99 (0.78–1.26)	0.98 (0.79–1.21)		
MACE overall	0.85 (0.66–1.01)	0.77 (0.63–0.97)	0.84 (0.68–1.07)	0.84 (0.70–1.00)	VKA
MI overall	0.68 (0.53–0.86)	0.83 (0.72–0.96)	0.85 (0.75–0.97)	0.84 (0.74–0.95)	
MACE ≥ 12 months	0.84 (0.59–1.09)	0.84 (0.57–1.28)	0.94 (0.61–1.51)	0.85 (0.63–1.15)	
MACE < 12 months	–	0.7 (0.02–30.06)	0.76 (0.02–32.72)	0.83 (0.02–32.72)	
MACE < 75 years	0.84 (0.55–1.15)	0.78 (0.45–1.43)	0.93 (0.54–1.69)	0.87 (0.6–1.25)	
MI ≥ 12 months	0.82 (0.68–0.99)	0.84 (0.75–0.95)	0.92 (0.82–1.03)	0.88 (0.0–0.97)	
MI < 12 months	0.45 (0.21–0.98)	0.98 (0.55–1.73)	0.76 (0.54–1.08)	0.74 (0.45–1.21)	
MI ≥ 75 years	0.80 (0.57–1.09)	0.87 (0.71–1.04)	0.92 (0.77–1.1)	0.85 (0.72–1.00)	
MI < 75 years	0.60 (0.41–0.85)	0.84 (0.67–1.06)	0.83 (0.69–1.0)	0.85 (0.69–1.04)	

B. RCTs					
Outcomes HR (95%CI)	Edoxaban				
MI overall	–	Apixaban			
MI < 75 years	–				
MI overall	–	–	Dabigatran		
MI < 75 years	–	–			
MI overall	–	–	1.19 (0.63–2.25)	Rivaroxaban	
MI < 75 years	–	–	1.2 (0.63–2.25)		
MI overall	–	–	1.23 (0.81–1.89)	1.03 (0.65–1.67)	VKA
MI < 75 years	–	–	1.23 (0.81–1.88)	1.03 (0.65–1.67)	

*CI* credible interval, *HR* hazard ratio, *MACE* major adverse cardiac events, *MI* myocardial infarction, *VKA* vitamin K antagonist

In the sub-group analysis (Table 2, Panel A), edoxaban (HR 0.84, 95%CI 0.68–0.99), rivaroxaban (HR 0.88, 95%CI 0.05–0.097), and apixaban (HR 0.84, 95%CI 0.75–0.95) were associated with a lower risk of MI during a median follow-up ≥ 12 months, with edoxaban associated with a lower MI risk also in patients with a follow-up < 12 months (HR 0.45, 95%CI 0.21–0.98) compared to VKA. No difference was observed according to age sub-analysis (Table 2). Most of our results were derived from direct comparison. However, to evaluate the agreement between direct and indirect evidence for MACE and MI in the analyses that did not include only the direct comparison, we performed coherence that did not differ between direct and indirect evidence as shown in Supplementary Fig. 7, Panel A and B.

### Meta-regression analysis

The meta-regression analysis showed that history of cardiovascular disease and the concomitant use of antiplatelets did not differ from main and sub-group analyses for MI and MACE as shown in Supplementary Table 4.

## Discussion

Our meta-analysis on a large sample of AF patients sought to investigate the best anticoagulant treatment to manage cardiovascular risk beyond thromboembolism. The principal finding of this large network meta-analysis is that DOACs reduce the risk of MI compared with VKAs, with apixaban also being associated with a reduced risk of MACE. This association was consistent across most sub-groups, with relatively low heterogeneity, thus reinforcing the robustness of the results.

We found that overall, all DOACs reduced the MI risk, especially for long-term treatments > 12 months. In addition, we found that edoxaban use was associated with a lower risk of MI compared to VKA in patients with a median follow-up < 12 months.

In addition, we found that edoxaban use was associated with a lower risk of MI compared to VKA in patients with a median follow-up < 12 months. This effect was not evident in the randomized ENTRUST-AF PCI trial [23] that had a median follow-up of 364 days. However, this trial was designed for major bleeding as principal endpoint, and despite the high annual rate of MI, the absolute number was low (< 30 events per group), and this might not be enough to detect any difference. Furthermore, patients had some clinical characteristics that may affect results, such as the lower mean age of patients (69–70 years) and only a minority were women (26%). Finally, > 15% in each treatment group prematurely discontinued treatment that in such short follow-up may be relevant.

Our results also showed that aging seems to be an important factor influencing the effectiveness of DOACs on cardiovascular outcomes. While there is evidence that DOACs are effective in reducing thromboembolic stroke in elderly AF patients [24], the effect on MI seems to be limited. Indeed, only a trend for rivaroxaban towards a reduction of MI risk was observed in the sub-group of patients aged 75 years or more. No evidence on elderly may be extrapolated from RCTs as the median age of patients was lower than observational ones.

Conversely, edoxaban showed a greater reduction of MI in patients < 75 years compared to VKA. This difference may rely on different clinical characteristics, concomitant drugs administration and clinical use of DOACs that are difficult to extrapolate by this analysis, as in all studies, DOAC prescription was driven by thromboembolic prophylaxis rather than cardiovascular risk assessment.

Considering the broader definition of MACE, which also includes cardiovascular mortality, we found that only apixaban and a trend for rivaroxaban were observed toward a reduction compared to VKAs.

Our results suggest that in addition to the proactive management of traditional modifiable cardiovascular risk factors, as suggested by current guidelines (according to the AF-CARE [1] or the ABC pathway [25]) and the prescription of drugs known to reduce cardiovascular risk (such as statins or SGLT2 inhibitors), the choice of the appropriate anticoagulant treatment may help to further reduce residual cardiovascular risk in this population, especially in elderly AF patients.

However, there are many factors that may have influenced our results, such as the optimization of cardioactive therapy, the varying standards of care in the years across which the studies were published, and the advancements in revascularization strategies.

### Biological plausibility

The effectiveness of DOACs in reducing atherothrombotic complications beyond thromboembolism may depend on different mechanisms, not all still explored. One possible explanation is that DOACs may exert direct and indirect antiplatelet effects beyond their anticoagulant action [26]. Overall, DOACs can reduce thrombin generation, a potent platelet agonist, and thromboxane production [26].

Rivaroxaban has been shown to reduce platelet activation by inhibiting platelet glycoprotein VI [27]; in addition, in platelets co-incubated with aspirin, rivaroxaban amplified the aspirin antiplatelet effect [27]. Recent evidence also showed that dabigatran inhibited thrombin binding to platelets possibly resulting in lower platelet activation [28]. DOACs may also delay platelet aggregation secondary to coagulation activation with a more potent effect seen with dabigatran [29].

In vitro study showed that apixaban at high concentration may reduce fibrin formation, lower platelet deposition, and delay clot formation [30]. Finally, thrombin-induced platelet aggregation is impaired in non-valvular AF patients treated with edoxaban [31].

### Strengths and limitations

The strength of this meta-analysis relies on the very large sample of patients included and high number of events for each endpoint and on the generally low heterogeneity, which make our results robust. Our results provide further data on the efficacy of DOACs in addition to the evidence from RCTs which allowed their marketing for the prevention of ischemic stroke in patients with AF. Thus, although phase III clinical trials provide the highest quality level of evidence, it is important to have results from observational real-life cohort studies, as patients in daily clinical practice may substantially differ from those included in RCTs, in terms of comorbidities as very elderly or patients with cancer or advanced chronic and liver disease are usually excluded or underrepresented. Furthermore, patients included in RCTs are usually strictly monitored and length of follow-up is usually limited. As a result, the reported risk of MI/MACE was lower in RCT compared to real-world observational studies [10].

This analysis has several limitations to acknowledge which are mainly related to the heterogeneous patients’ enrollment and to the use of network meta-analysis. Given the lack of head-to-head trials comparing the safety and efficacy of DOACs, these results should be intended as indirect comparisons with some possible missing confounding factors not considered.

To partially overcome this limitation, we retrieved from each study the HR for each outcome derived from the multivariable adjusted Cox proportional hazard regression analysis or from propensity score matching.

However, despite most observational studies using the inverse probability of treatment weighting or propensity score matching to minimize confounding, the risk of residual confounding still persists.

In addition, potential publication bias should be addressed. Finally, we included only studies that compared DOACs and VKAs; however, after DOACs introduction, VKAs are reserved for specific populations such as patients with advanced chronic kidney disease or recurrent cerebrovascular events, and this could represent a confounding factor in our analysis.

In conclusion, specific DOACs may offer incremental benefits for cardiovascular outcomes beyond stroke prevention in AF. A tailored approach taking into account the global cardiovascular risk of AF patients is needed given the significant residual cardiovascular risk of this patients’ population.

## Supplementary Information

Below is the link to the electronic supplementary material.

Supplementary file1 (DOCX 5387 KB)
