# Supplementary material for: Effects of direct oral anticoagulants on cardiac outcomes in atrial fibrillation: a systematic review and network meta-analysis
Source: Intern Emerg Med. 2026 Aug 3;21(6):2305–20. doi: 10.1007/s11739-026-04480-1 (PMC13582121; doi:10.1007/s11739-026-04480-1)
Supplement: Supplementary file 1 [file 11739_2026_4480_MOESM1_ESM.docx]

**Effects of direct oral anticoagulants on cardiac outcomes in atrial fibrillation: a systematic review and network meta-analysis.**

*Supplementary data*

**Supplement S1 Search strategy.**

**Supplementary Table 1. PRISMA checklist**

**Supplementary Table 2. Proportion of each anticoagulant in studies included.**

**Supplementary Table 3. Confounders evaluated for each study included in the meta-analysis.**

**Supplementary Table 4. Meta-regression analysis of clinical endpoints according to antiplatelet use and previous cardiovascular disease.**

**Supplementary Figure 1. Network according to Major Adverse Clinical Events (MACE): in all patients with atrial fibrillation (AF) alone (Panel A), in all patients with AF undergoing percutaneous coronary artery intervention (PCI) (Panel B), in patients <75 years with AF alone (Panel C), in patients with AF alone and a median follow-up ≥12 months (Panel D), in patients with AF alone and a median follow-up <12 months (Panel E).**

**Supplementary Figure 2. Network according to Myocardial Infarction (MI): in all patients with atrial fibrillation (AF) alone (Panel A), in all patients with AF undergoing percutaneous coronary artery intervention (PCI) in clinical trials (Panel B), in all patients with AF undergoing PCI in observational studies (Panel C), in patients<75 years with AF undergoing PCI (Panel D), in patients <75 years with AF alone (E), in patients ≥75 years with AF alone (F), in patients with AF alone and a median follow-up <12 months (G), in patients with AF alone and a median follow-up ≥12 months (H).**

**Supplementary Figure 3. PRISMA flowchart diagram.**

**Supplementary Figure 4. Risk of bias assessment: A. Summary plot RoB 2.0 for RCT; B. Traffic light plot RoB 2.0 for RCT; C. Summary plot ROBINS -I for observational studies; D. Traffic light plot ROBINS-I for observational studies.**

**Supplementary Figure 5. Funnel plots for publication bias for major adverse cardiac events (MACE). MACE in patients with atrial fibrillation (AF) alone (Panel A), MACE in patients with a median follow-up ≥ 12 months (Panel B), MACE in patients with a median follow-up < 12 months (Panel C), MACE in patients < 75 years with AF alone (Panel D), MACE in patients with AF undergoing percutaneous coronary intervention (PCI) (Panel E).**

**Supplementary Figure 6. Funnel plots for publication bias for myocardial infarction (MI). MI in patients with atrial fibrillation (AF) undergoing percutaneous coronary interventions (PCI), only clinical trials (Panel A), and only observational studies (Panel B). MI in patients < 75 years with AF and PCI (Panel C), MI in patients with AF alone (Panel D), MI in patients with AF alone and a median follow-up ≥ 12 months (Panel E), MI in patients with AF alone and a median follow-up < 12 months (Panel F), MI in patients ≥ 75 years with AF alone (Panel G) MI in patients < 75 years with AF alone (H).**

**Supplementary Figure 7. Coherence between direct and indirect evidence for major adverse cardiac events (MACE) (A) and myocardial infarction (MI) (B).**

**Supplement S1**

**Search strategy.**

*PubMed (Medline)*

(((((((((DOAC[Title/Abstract]) OR (NOAC[Title/Abstract])) OR (rivaroxaban[Title/Abstract])) OR (edoxaban[Title/Abstract])) OR (dabigatran[Title/Abstract])) OR (apixaban[Title/Abstract])) AND ((myocardial infarction[Title/Abstract]) OR (AMI[Title/Abstract]) OR (Heart Attack[MeSH Terms]) OR (major adverse cardiac events[Title/Abstract]) OR (MACE[Title/Abstract]))

**Supplementary Table 1. PRISMA checklist**

| **Section and Topic** | **Item #** | **Checklist item** | **Location where item is reported** |
| --- | --- | --- | --- |
| **TITLE** | | |  |
| Title | 1 | Identify the report as a systematic review. | Pag. 1 |
| **ABSTRACT** | | |  |
| Abstract | 2 | See the PRISMA 2020 for Abstracts checklist. | Pag. 2 |
| **INTRODUCTION** | | |  |
| Rationale | 3 | Describe the rationale for the review in the context of existing knowledge. | Pag. 4-5 |
| Objectives | 4 | Provide an explicit statement of the objective(s) or question(s) the review addresses. | Pag. 4-5 |
| **METHODS** | | |  |
| Eligibility criteria | 5 | Specify the inclusion and exclusion criteria for the review and how studies were grouped for the syntheses. | Pag. 5 |
| Information sources | 6 | Specify all databases, registers, websites, organisations, reference lists and other sources searched or consulted to identify studies. Specify the date when each source was last searched or consulted. | Pag. 5 + Supplementary |
| Search strategy | 7 | Present the full search strategies for all databases, registers, and websites, including any filters and limits used. | Supplementary S1 |
| Selection process | 8 | Specify the methods used to decide whether a study met the inclusion criteria of the review, including how many reviewers screened each record and each report retrieved, whether they worked independently, and if applicable, details of automation tools used in the process. | Pag. 5-6 |
| Data collection process | 9 | Specify the methods used to collect data from reports, including how many reviewers collected data from each report, whether they worked independently, any processes for obtaining or confirming data from study investigators, and if applicable, details of automation tools used in the process. | Pag. 6-7 |
| Data items | 10a | List and define all outcomes for which data were sought. Specify whether all results that were compatible with each outcome domain in each study were sought (e.g. for all measures, time points, analyses), and if not, the methods used to decide which results to collect. | Pag. 5-6 |
|  | 10b | List and define all other variables for which data were sought (e.g. participant and intervention characteristics, funding sources). Describe any assumptions made about any missing or unclear information. | Pag. 5-6 |
| Study risk of bias assessment | 11 | Specify the methods used to assess risk of bias in the included studies, including details of the tool(s) used, how many reviewers assessed each study and whether they worked independently, and if applicable, details of automation tools used in the process. | Pag. 6 |
| Effect measures | 12 | Specify for each outcome the effect measure(s) (e.g. risk ratio, mean difference) used in the synthesis or presentation of results. | Pag. 6-7 |
| Synthesis methods | 13a | Describe the processes used to decide which studies were eligible for each synthesis (e.g. tabulating the study intervention characteristics and comparing against the planned groups for each synthesis (item #5)). | Pag. 5 |
|  | 13b | Describe any methods required to prepare the data for presentation or synthesis, such as handling of missing summary statistics, or data conversions. | Pag. 6-7 |
|  | 13c | Describe any methods used to tabulate or visually display results of individual studies and syntheses. | Pag. 6-7 |
|  | 13d | Describe any methods used to synthesize results and provide a rationale for the choice(s). If meta-analysis was performed, describe the model(s), method(s) to identify the presence and extent of statistical heterogeneity, and software package(s) used. | Pag. 6-7 |
|  | 13e | Describe any methods used to explore possible causes of heterogeneity among study results (e.g. subgroup analysis, meta-regression). | Pag. 6-7 |
|  | 13f | Describe any sensitivity analyses conducted to assess robustness of the synthesized results. | Pag. 6-7 |
| Reporting bias assessment | 14 | Describe any methods used to assess risk of bias due to missing results in a synthesis (arising from reporting biases). | Pag. 6-7 |
| Certainty assessment | 15 | Describe any methods used to assess certainty (or confidence) in the body of evidence for an outcome. | Pag. 6-7 |
| **RESULTS** | | |  |
| Study selection | 16a | Describe the results of the search and selection process, from the number of records identified in the search to the number of studies included in the review, ideally using a flow diagram. | Pag. 7 |
|  | 16b | Cite studies that might appear to meet the inclusion criteria, but which were excluded, and explain why they were excluded. | Pag. 7 |
| Study characteristics | 17 | Cite each included study and present its characteristics. | Pag. 7-8 |
| Risk of bias in studies | 18 | Present assessments of risk of bias for each included study. | Pg. 8 + Supplementary Fig 3 |
| Results of individual studies | 19 | For all outcomes, present, for each study: (a) summary statistics for each group (where appropriate) and (b) an effect estimate and its precision (e.g. confidence/credible interval), ideally using structured tables or plots. | Pag. 8-9 |
| Results of syntheses | 20a | For each synthesis, briefly summarise the characteristics and risk of bias among contributing studies. | Pag. 9 |
|  | 20b | Present results of all statistical syntheses conducted. If meta-analysis was done, present for each the summary estimate and its precision (e.g. confidence/credible interval) and measures of statistical heterogeneity. If comparing groups, describe the direction of the effect. | Pag. 9 |
|  | 20c | Present results of all investigations of possible causes of heterogeneity among study results. | Pag. 9 |
|  | 20d | Present results of all sensitivity analyses conducted to assess the robustness of the synthesized results. | Pag. 9 |
| Reporting biases | 21 | Present assessments of risk of bias due to missing results (arising from reporting biases) for each synthesis assessed. | Pag. 9 |
| Certainty of evidence | 22 | Present assessments of certainty (or confidence) in the body of evidence for each outcome assessed. | Pag. 9 |
| **DISCUSSION** | | |  |
| Discussion | 23a | Provide a general interpretation of the results in the context of other evidence. | Pag. 10 |
|  | 23b | Discuss any limitations of the evidence included in the review. | Pag. 12 |
|  | 23c | Discuss any limitations of the review processes used. | Pag. 12 |
|  | 23d | Discuss implications of the results for practice, policy, and future research. | Pag. 12 |
| **OTHER INFORMATION** | | |  |
| Registration and protocol | 24a | Provide registration information for the review, including register name and registration number, or state that the review was not registered. | Pag. 2 |
|  | 24b | Indicate where the review protocol can be accessed, or state that a protocol was not prepared. | Pag. 2 |
|  | 24c | Describe and explain any amendments to information provided at registration or in the protocol. | Pag.9-10 |
| Support | 25 | Describe sources of financial or non-financial support for the review, and the role of the funders or sponsors in the review. | 1 |
| Competing interests | 26 | Declare any competing interests of review authors. | 1 |
| Availability of data, code and other materials | 27 | Report which of the following are publicly available and where they can be found: template data collection forms; data extracted from included studies; data used for all analyses; analytic code; any other materials used in the review. | N/A |

**Supplementary Table 2. Proportion of each anticoagulant in studies included.**

| **Year** | **First Author /Cohort** | **Design** | **Total Cohort** | **Dabigatran** | **Apixaban** | **Rivaroxaban** | **Edoxaban** | **VKA** |
| --- | --- | --- | --- | --- | --- | --- | --- | --- |
| 2013 | ENGAGE-AF TIMI 48[1] | PostH | 21105 | - | - | - | 14069 | 7036 |
| 2021 | A. Briasoulis[2] | R | 28011 | 4233 | 6052 | 4309 | - | 13417 |
| 2021 | R. D. Lopes[3] | R | 155038 | 9347 | 34066 | 45222 |  | 66403 |
| 2021 | A. Elis[4] | P | 252 | - | 97 | - | - | 155 |
| 2021 | E. Crocetti[5] | R | 8543 | 1636 | 2693 | 1385 | 1725 | 1104 |
| 2022 | P. Liu[6] | R | 1246 | - | - | 787 | - | 459 |
| 2022 | GLORIA-AF[7] | P | 12359 | 3839 | 4505 | 4015 | - | - |
| 2022 | L. Boivin-Proulx[8] | R | 1656 | - | - | 403 | - | 1253 |
| 2022 | L. Boivin-Proulx[8] | R | 1792 | - | 539 | - | - | 1253 |
| 2022 | Q. Yu[9] | R | 319 | - | - | 178 | - | 141 |
| 2022 | A. Jaksa[10] | R | 5655 | 261 | 2801 | 2221 | 398 | - |
| 2023 | Y. Talmor-Barkan[11] | R | 56553 | 5770 | 35101 | 15682 | - | - |
| 2021 | A. B. Ingason[12] | R | 4670 | 420 | 1787 | 2463 | - | - |
| 2023 | M. Grymonprez[13] | R | 254478 | 28144 | 66925 | 74421 | 23582 | 61406 |
| 2021 | R. D. Lopes[14] | R | 94435 | 6966 | 15616 | 27209 | - | 44644 |
| 2020 | Chan[15] | R | 26779 | 6531 | 3249 | 9798 | 1389 | 5811 |
| 2019 | Pratt[16] | R | 4836 | 627 | 1127 | 1718 | - | 1364 |
| 2019 | Amin[17] | R | 198171 | 18162 | 38466 | 60133 | - | 81410 |
| 2019 | Russo[18] | P | 899 | 383 | - | - | - | 516 |
| 2018 | Yu[19] | R | 11712 | - | - | - | 5856 | 5856 |
| 2018 | Lai[20] | R | 4722 | 1489 | - | 1736 | - | 1496 |
| 2018 | Lee[21] | R | 31739 | 7377 | 8611 | 6838 | - | 8913 |
| 2018 | Barnett (ROCKET AF) [22] | PostH | 14236 | - | - | 7111 | - | 7125 |
| 2020 | Coleman[23] | R | 8303 | - | - | 3257 | - | 5046 |
| 2019 | Baker[24] | R | 24646 | - | - | 10700 | - | 13946 |
| 2018 | Själander[25] | R | 64382 | 6574 | 12311 | 8323 | - | 37174 |
| 2018 | Briasoulis[26] | R | 85596 | 13522 | - | 14257 | - | 57817 |
| 2018 | Hsin-Fu Lee[27] | R | 42000 | - | - | 26000 | - | 16000 |
| 2016 | Gibson  (PIONER-AF) [28] | RCT | 2124 | - | - | 709 | - | 706 |
| 2018 | Mueller[29] | R | 14577 | 1112 | 6200 | 7265 | - | - |
| 2020 | Vranckx (ENTRUST-AF PCI trial[30] | RCT | 1506 | - | - | - | 751 | 755 |
| 2019 | Oldgren (RE-DUAL PCI trial) [31] | RCT | 2725 | 1744 | - | - | - | 981 |
| 2012 | Hohnloser (RE-LY) [32] | PostH | 18113 | - | - | - | 12091 | 6022 |
| 2013 | Bahit (ARISTOTLE) [33] | PostH | 18184 | - | 9092 | - | - | 9092 |
| 2014 | Larsen[34] | R | 13914 | 4978 | - | - | - | 8936 |
| 2015 | Graham[35] | R | 134414 | - | - | - | 67207 | 67207 |
| 2015 | Lauffenburger[36] | R | 64935 | - | - | - | 21070 | 43865 |
| 2015 | Seeger - MARKETSCAN[37] | R | 31058 | 15529 | - | - | - | 15529 |
| 2015 | Seeger - Clinformatics[37] | R | 7320 | 3660 | - | - | - | 3660 |
| 2015 | Chan[38] | R | 19853 | 9940 | - | - | - | 9913 |
| 2015 | Villines[39] | R | 25586 | 12793 | - | - | - | 12793 |
| 2017 | Coleman[40] | R | 1670 | - | 835 | - | - | 835 |
| 2017 | Go[41] | R | 50578 | 25289 | - | - | - | 25289 |
| 2017 | Lai[42] | R | 9200 | 4600 | - | 4600 | - | - |
| 2017 | Norby[43] | R | 33914 | 16957 | - | 16957 | - | - |
| 2017 | Norby[43] | R | 77991 | - | - | 32495 | - | 45496 |
| 2017 | Shantha[44] | R | 33111 | 11037 | - | 11037 | - | 11037 |
| 2017 | Shantha[44] | R | 22827 | 7609 | - | 7609 | - | 7609 |
| 2024 | Burnham[45] | R | 333 | - | 212 | 121 | - | - |
| 2023 | Pereira[46] | P | 53 | - | - | 27 | - | 26 |
| 2025 | Dari[47] | R | 16160 | - | 9990 | 6170 | - | - |
| 2024 | Kreutz[48] | P | 1455 | - | - | 764 | - | 691 |
| 2024 | Liu[49] | R | 1301 | 514 | - | 787 | - | - |
| 2025 | Fang[50] | R | 2952 | - | - | - | - | 1476 |

**Supplementary Table 3. Confounders evaluated for each study included in the meta-analysis.**

| **Year** | **First Author /Cohort** | **Confounders evaluated** |  |  |
| --- | --- | --- | --- | --- |
| 2012 | Hohnloser (RE-LY) | RCT – N/A |  |  |
| 2013 | ENGAGE-AF TIMI 48 | RCT – N/A |  |  |
| 2013 | Bahit (ARISTOTLE) | RCT – N/A |  |  |
| 2014 | Larsen | PSM – adjusted for all baseline characteristics |  |  |
| 2015 | Graham | PSM – adjusted for all baseline characteristics | Lauffenburger | PSM – adjusted for all baseline characteristics |
| 2015 | Lauffenburger | PSM – adjusted for all baseline characteristics |  |  |
| 2015 | Seeger - MARKETSCAN | PSM – adjusted for pre-specified 78 covariates |  |  |
| 2015 | Seeger - Clinformatics | PSM – adjusted for pre-specified 78 covariates |  |  |
| 2015 | Chan | IPTW – adjusted for all baseline characteristics |  |  |
| 2015 | Villines | PSM – adjusted for all baseline characteristics |  |  |
| 2016 | Gibson  (PIONER-AF) | RCT – N/A |  |  |
| 2017 | Coleman | PSM – adjusted for all baseline characteristics |  |  |
| 2017 | Go | PSM – adjusted for all baseline characteristics |  |  |
| 2017 | Lai | PSM – adjusted for all baseline characteristics |  |  |
| 2017 | Norby | PSM – adjusted for all baseline characteristics |  |  |
| 2017 | Norby | PSM – adjusted for all baseline characteristics |  |  |
| 2017 | Shantha | PSM – adjusted for all baseline characteristics |  |  |
| 2017 | Shantha | PSM – adjusted for all baseline characteristics |  |  |
| 2018 | Yu | PSM – adjusted for all baseline characteristics |  |  |
| 2018 | Lai | PSM – adjusted for all baseline characteristics |  |  |
| 2018 | Lee | oral anticoagulation treatment, sex, heart failure, diabetes, hypertension, vascular disease, liver disease, stroke, and bleeding |  |  |
| 2018 | Barnett  (ROCKET AF) | RCT – N/A |  |  |
| 2018 | Själander | PSM – adjusted for all baseline characteristics |  |  |
| 2018 | Briasoulis | PSM – adjusted for all baseline characteristics |  |  |
| 2018 | Hsin-Fu Lee | PSM – adjusted for all baseline characteristics |  |  |
| 2018 | Mueller | Sex, age, Charlson Score, CHA_2_DS_2_-VASc, HASBLED, polypharmacy |  |  |
| 2019 | Pratt | IPTW – adjusted for all baseline characteristics |  |  |
| 2019 | Amin | PSM – adjusted for all baseline characteristics |  |  |
| 2019 | Russo | PSM – adjusted for all baseline characteristics |  |  |
| 2019 | Baker | IPTW – adjusted for all baseline characteristics |  |  |
| 2019 | Oldgren  (RE-DUAL PCI trial) | RCT – N/A |  |  |
| 2020 | Chan | PSM – adjusted for all baseline characteristics |  |  |
| 2020 | Coleman | IPTW – adjusted for all baseline characteristics |  |  |
| 2020 | Vranckx (ENTRUST-AF PCI trial) | RCT – N/A |  |  |
| 2021 | A. B. Ingason | PSM – adjusted for all baseline characteristics |  |  |
| 2021 | A. Briasoulis | IPTW – adjusted for all baseline characteristics |  |  |
| 2021 | R. D. Lopes | PSM – adjusted for all baseline characteristics |  |  |
| 2021 | A. Elis | PSM – adjusted for all baseline characteristics |  |  |
| 2021 | E. Crocetti | PSM – adjusted for all baseline characteristics |  |  |
| 2021 | R. D. Lopes | PSM – adjusted for all baseline characteristics |  |  |
| 2022 | P. Liu | Multivariable covariates were not reported |  |  |
| 2022 | GLORIA-AF | PSM – adjusted for all baseline characteristics |  |  |
| 2022 | L. Boivin-Proulx | IPTW – adjusted for all baseline characteristics |  |  |
| 2022 | L. Boivin-Proulx | IPTW – adjusted for all baseline characteristics |  |  |
| 2022 | Q. Yu | Age, sex, CHA_2_DS_2_-VASc score, HAS-BLED score, Duration, Diagnosis, Lesion characteristics, Lesion length, Thrombus Debulking, Critical limb ischemia, History of index-limb revascularization, Hypertension, Diabetes mellitus, Smoking status, Chronic Renal failure, Pre-procedural medication, Coronary artery disease, Ischemic stroke, Chronic Renal failure, Pre-procedural medication, Post-procedural medication, Pre-procedural anticoagulant, Post-procedural anticoagulant |  |  |
| 2022 | A. Jaksa | PSM – adjusted for all baseline characteristics |  |  |
| 2023 | Y. Talmor-Barkan | PSM – adjusted for all baseline characteristics |  |  |
| 2023 | M. Grymonprez | PSM – adjusted for all baseline characteristics |  |  |
| 2023 | Pereira | Not reported |  |  |
| 2024 | Burnham | Not reported |  |  |
| 2024 | Kreutz | PSM – adjusted for all baseline characteristics |  |  |
| 2024 | Liu | Multivariable covariates were not reported |  |  |
| 2025 | Dari | PSM – adjusted for all baseline characteristics |  |  |
| 2025 | Fang | PSM – adjusted for all baseline characteristics |  |  |

*DAPT: dual antiplatelet, IPTW: inverse probability of treatment weighting, N/A: not applicable, PSM: propensity score matching, RCT: randomized clinical trial,*

**Supplementary Table 4. Meta-regression analysis of clinical endpoints according to antiplatelet use and previous cardiovascular disease.**

| A. Myocardial infarction | Cov (log HR) | effect | 95%CI | |
| --- | --- | --- | --- | --- |
| All AF patients undergoing PCI | pCVD | 0.626 | -1.477 | 2.587 |
| All AF patients undergoing PCI | Antiplatelets | 0.222 | -0.553 | 0.956 |
| AF patients < 75 years undergoing PCI | pCVD | 0.621 | -1.495 | 2.595 |
| AF patients < 75 years undergoing PCI | Antiplatelets | 0.220 | -0.552 | 0.962 |
| AF patients alone | pCVD | -0.006 | -0.238 | 0.217 |
| AF patients alone | Antiplatelets | -0.074 | -0.300 | 0.148 |
| AF patients with a median follow-up ≥12 months | pCVD | -0.046 | -0.306 | 0.202 |
| AF patients with a median follow-up ≥12 months | Antiplatelets | 0.094 | -0.052 | 0.238 |
| AF patients with a median follow-up <12 months | pCVD | 0.130 | -0.571 | 0.796 |
| AF patients with a median follow-up <12 months | Antiplatelets | -0.517 | -1.660 | 0.703 |
| AF patients < 75 years | pCVD | 0.076 | -0.200 | 0.351 |
| AF patients < 75 years | Antiplatelets | -0.212 | -0.630 | 0.189 |
| AF patients ≥ 75 years | pCVD | -0.189 | -0.744 | 0.324 |
| AF patients ≥ 75 years | Antiplatelets | 0.040 | -0.174 | 0.237 |
| B. Major adverse cardiac events (MACE) | **Cov (log HR)** | **effect** | **95%CI** | |
| AF patients overall | pCVD | 0.242 | -0.570 | 1.007 |
| AF patients overall | Antiplatelets | 0.755 | -0.182 | 1.654 |
| AF patients < 75 years | pCVD | 0.180 | -1.909 | 2.993 |
| AF patients < 75 years | Antiplatelets | 0.304 | -0.449 | 1.046 |
| AF patients with a median follow-up ≥12 months | pCVD | 0.177 | -1.851 | 2.656 |
| AF patients with a median follow-up ≥12 months | Antiplatelets | 0.307 | -0.445 | 1.077 |

*AF: atrial fibrillation, CI: confidence interval, MI: myocardial infarction, PCI: percutaneous coronary intervention, pCVD: previous cardiovascular disease.*

**Supplementary Figure 1. Network according to Major Adverse Clinical Events (MACE): in all patients with atrial fibrillation (AF) (Panel A), in patients <75 years with AF (Panel B), in patients with AF and a median follow-up ≥12 months (Panel C), in patients with AF and a median follow-up <12 months (Panel D).**

**A	B**

**C	D**

*Api: apixaban, Dab: dabigatran, Edo: edoxaban, Riva: rivaroxaban, VKA: vitamin K antagonist*

**Supplementary Figure 2. Network according to Myocardial Infarction (MI): in all patients with atrial fibrillation (AF) alone (Panel A), in all patients with AF undergoing percutaneous coronary artery intervention (PCI) in clinical trials (Panel B), in all patients with AF undergoing PCI in observational studies (Panel C), in patients<75 years with AF undergoing PCI (Panel D), in patients <75 years with AF alone (E), in patients ≥75 years with AF alone (F), in patients with AF alone and a median follow-up <12 months (G), in patients with AF alone and a median follow-up ≥12 months (H).**

**A	B	C**

**D	E	F**

**G	H**

*Api: apixaban, Dab: dabigatran, Edo: edoxaban, Riva: rivaroxaban, VKA: vitamin K antagonist*

**Supplementary Figure 3. PRISMA flowchart diagram.**

**Identification of studies via databases and registers**

Records identified from:

PubMed (n = 1,304)

**Identification**

Records excluded

(n = 1,155)

Records screened

(n = 1,304)

Reports excluded:

Meta-analysis/review (n = 35)

Not addressing study question (n =24)

No control group (n =5)

No data about outcome of interest (n: 35)

**Screening**

Reports assessed for eligibility

(n = 149)

**Included**

Studies included in the meta-analysis

(n = 50)

**Supplementary Figure 4. Risk of bias assessment: A Summary plot RoB 2.0 for RCT; B Traffic light plot RoB 2.0 for RCT; C Summary plot ROBINS -I for observational studies; D Traffic light plot ROBINS-I for observational studies.**

**A**

**B**

**C**

**D**

**Supplementary Figure 5. Funnel plots for publication bias for major adverse cardiac events (MACE). MACE in all patients with atrial fibrillation (AF) (Panel A), MACE in patients with a median follow-up ≥ 12 months (Panel B), MACE in patients with a median follow-up < 12 months (Panel C), MACE in patients < 75 years with AF alone (Panel D).**

**A	B	C**

**D**

**Supplementary Figure 6. Funnel plots for publication bias for myocardial infarction (MI). MI in patients with atrial fibrillation (AF) undergoing percutaneous coronary interventions (PCI), only clinical trials (Panel A), and only observational studies (Panel B). MI in patients < 75 years with AF and PCI (Panel C), MI in patients with AF alone (Panel D), MI in patients with AF alone and a median follow-up ≥ 12 months (Panel E), MI in patients with AF alone and a median follow-up < 12 months (Panel F), MI in patients ≥ 75 years with AF alone (Panel G) MI in patients < 75 years with AF alone (H).**

**A	B	C**

**D	E	F**

**G	H**

**Supplementary Figure 7. Coherence between direct and indirect evidence for major adverse cardiac events (MACE) (A) and myocardial infarction (MI) (B).**

**A. Overall AF patients**

**AF patients <75 years**

**AF patients with a median follow-up ≥12 months**

**C. Overall AF patients**

**AF patients <75 years**

**AF patients ≥75 years**

**AF patients with a median follow-up <12 months**

**AF patients with a median follow-up ≥12 months**

*AF: atrial fibrillation*

**Reference**

1. Vilain K, Li H, Kwong WJ, Antman EM, Ruff CT, Braunwald E, Cohen DJ, Giugliano RP, Magnuson EA, Investigators EA-TT (2020) Cardiovascular- and Bleeding-Related Hospitalization Rates With Edoxaban Versus Warfarin in Patients With Atrial Fibrillation Based on Results of the ENGAGE AF-TIMI 48 Trial. Circ Cardiovasc Qual Outcomes 13:e006511. <https://doi.org/10.1161/CIRCOUTCOMES.120.006511>

2. Briasoulis A, Mentias A, Mazur A, Alvarez P, Leira EC, Vaughan Sarrazin MS (2021) Comparative Effectiveness and Safety of Direct Oral Anticoagulants in Obese Patients with Atrial Fibrillation. Cardiovasc Drugs Ther 35:261-272. <https://doi.org/10.1007/s10557-020-07126-2>

3. Lopes RD, Thomas L, Di Fusco M, Keshishian A, Luo X, Li X, Masseria C, Friend K, Mardekian J, Pan X, Yuce H, Jones WS (2021) Clinical and Economic Outcomes Among Nonvalvular Atrial Fibrillation Patients With Coronary Artery Disease and/or Peripheral Artery Disease. Am J Cardiol 148:69-77. <https://doi.org/10.1016/j.amjcard.2021.02.021>

4. Elis A, Klempfner R, Gurevitz C, Gilady E, Goldenberg I (2021) Apixaban in Patients with Atrial Fibrillation and Severe Renal Dysfunction: Findings from a National Registry. Isr Med Assoc J 23:353-358.

5. Crocetti E, Cattaneo S, Bergamaschi W, De Servi S, Russo AG (2021) Effectiveness and Safety of Non-Vitamin K Oral Anticoagulants in Non-Valvular Atrial Fibrillation Patients: Results of A Real-World Study in a Metropolitan Area of Northern Italy. J Clin Med 10. <https://doi.org/10.3390/jcm10194536>

6. Liu PH, Liu ZH, Niu MH, Chen P, Shi YB, He F, Guo R (2022) A Comparative Study of the Clinical Benefits of Rivaroxaban and Warfarin in Patients With Non-valvular Atrial Fibrillation With High Bleeding Risk. Front Cardiovasc Med 9:803233. <https://doi.org/10.3389/fcvm.2022.803233>

7. Lip GYH, Kotalczyk A, Teutsch C, Diener HC, Dubner SJ, Halperin JL, Ma CS, Rothman KJ, Marler S, Gurusamy VK, Huisman MV, Investigators G-A (2022) Comparative effectiveness and safety of non-vitamin K antagonists for atrial fibrillation in clinical practice: GLORIA-AF Registry. Clin Res Cardiol 111:560-573. <https://doi.org/10.1007/s00392-022-01996-2>

8. Boivin-Proulx LA, Potter BJ, Dorais M, Perreault S (2022) Comparative Effectiveness and Safety of Direct Oral Anticoagulants vs Warfarin Among Obese Patients With Atrial Fibrillation. CJC Open 4:395-405. <https://doi.org/10.1016/j.cjco.2022.01.002>

9. Yu Q, Chen C, Xu J, Xiao Y, Bao J, Yuan L (2022) Efficacy and safety evaluation of rivaroxaban vs. warfarin among non-valvular atrial fibrillation patients undergoing lower extremity revascularization. Front Cardiovasc Med 9:978639. <https://doi.org/10.3389/fcvm.2022.978639>

10. Jaksa A, Gibbs L, Kent S, Rowark S, Duffield S, Sharma M, Kincaid L, Ali AK, Patrick AR, Govil P, Jonsson P, Gatto N (2022) Using primary care data to assess comparative effectiveness and safety of apixaban and rivaroxaban in patients with nonvalvular atrial fibrillation in the UK: an observational cohort study. BMJ Open 12:e064662. <https://doi.org/10.1136/bmjopen-2022-064662>

11. Talmor-Barkan Y, Yacovzada NS, Rossman H, Witberg G, Kalka I, Kornowski R, Segal E (2022) Head-to-head efficacy and safety of rivaroxaban, apixaban, and dabigatran in an observational nationwide targeted trial. Eur Heart J Cardiovasc Pharmacother 9:26-37. <https://doi.org/10.1093/ehjcvp/pvac063>

12. Ingason AB, Hreinsson JP, Agustsson AS, Lund SH, Rumba E, Palsson DA, Reynisson IE, Gudmundsdottir BR, Onundarson PT, Bjornsson ES (2023) Comparison of the effectiveness and safety of direct oral anticoagulants: a nationwide propensity score-weighted study. Blood Adv 7:2564-2572. <https://doi.org/10.1182/bloodadvances.2022009099>

13. Grymonprez M, De Backer TL, Bertels X, Steurbaut S, Lahousse L (2023) Long-term comparative effectiveness and safety of dabigatran, rivaroxaban, apixaban and edoxaban in patients with atrial fibrillation: A nationwide cohort study. Front Pharmacol 14:1125576. <https://doi.org/10.3389/fphar.2023.1125576>

14. Lopes RD, Steffel J, Di Fusco M, Keshishian A, Luo X, Li X, Masseria C, Hamilton M, Friend K, Gupta K, Mardekian J, Pan X, Baser O, Jones WS (2020) Corrigendum to "Effectiveness and Safety of Oral Anticoagulants in Adults with Non-valvular Atrial Fibrillation Patients and Concomitant Coronary/Peripheral Artery Disease" American Journal of Medicine 131:09 (2018): 1074-1085.e4. Am J Med 133:1229-1238. <https://doi.org/10.1016/j.amjmed.2020.06.003>

15. Chan YH, Lee HF, Li PR, Liu JR, Chao TF, Wu LS, Chang SH, Yeh YH, Kuo CT, See LC, Lip GYH (2020) Effectiveness, safety, and major adverse limb events in atrial fibrillation patients with concomitant diabetes mellitus treated with non-vitamin K antagonist oral anticoagulants. Cardiovasc Diabetol 19:63. <https://doi.org/10.1186/s12933-020-01043-2>

16. Pratt NL, Ramsay E, Kalisch Ellett LM, Duszynski K, Shakib S, Kerr M, Caughey G, Roughead EE (2019) Comparative effectiveness and safety of low-strength and high-strength direct oral anticoagulants compared with warfarin: a sequential cohort study. BMJ Open 9:e026486. <https://doi.org/10.1136/bmjopen-2018-026486>

17. Amin A, Keshishian A, Dina O, Dhamane A, Nadkarni A, Carda E, Russ C, Rosenblatt L, Mardekian J, Yuce H, Baker CL (2019) Comparative clinical outcomes between direct oral anticoagulants and warfarin among elderly patients with non-valvular atrial fibrillation in the CMS medicare population. J Thromb Thrombolysis 48:240-249. <https://doi.org/10.1007/s11239-019-01838-5>

18. Russo V, Rago A, Proietti R, Attena E, Rainone C, Crisci M, Papa AA, Calabro P, D'Onofrio A, Golino P, Nigro G (2019) Safety and Efficacy of Triple Antithrombotic Therapy with Dabigatran versus Vitamin K Antagonist in Atrial Fibrillation Patients: A Pilot Study. Biomed Res Int 2019:5473240. <https://doi.org/10.1155/2019/5473240>

19. Yu HT, Yang PS, Kim TH, Jang E, Kim D, Uhm JS, Kim JY, Pak HN, Lee MH, Lip GYH, Joung B (2018) Impact of Renal Function on Outcomes With Edoxaban in Real-World Patients With Atrial Fibrillation. Stroke 49:2421-2429. <https://doi.org/10.1161/STROKEAHA.118.021387>

20. Lai CL, Chen HM, Liao MT, Lin TT (2018) Dabigatran, Rivaroxaban, and Warfarin in the Oldest Adults with Atrial Fibrillation in Taiwan. J Am Geriatr Soc 66:1567-1574. <https://doi.org/10.1111/jgs.15430>

21. Lee CJ, Gerds TA, Carlson N, Bonde AN, Gislason GH, Lamberts M, Olesen JB, Pallisgaard JL, Hansen ML, Torp-Pedersen C (2018) Risk of Myocardial Infarction in Anticoagulated Patients With Atrial Fibrillation. J Am Coll Cardiol 72:17-26. <https://doi.org/10.1016/j.jacc.2018.04.036>

22. Barnett AS, Cyr DD, Goodman SG, Levitan BS, Yuan Z, Hankey GJ, Singer DE, Becker RC, Breithardt G, Berkowitz SD, Halperin JL, Hacke W, Mahaffey KW, Nessel CC, Fox KAA, Patel MR, Piccini JP (2018) Net clinical benefit of rivaroxaban compared with warfarin in atrial fibrillation: Results from ROCKET AF. Int J Cardiol 257:78-83. <https://doi.org/10.1016/j.ijcard.2017.06.110>

23. Coleman CI, Baker WL, Meinecke AK, Eriksson D, Martinez BK, Bunz TJ, Alberts MJ (2020) Effectiveness and safety of rivaroxaban vs. warfarin in patients with non-valvular atrial fibrillation and coronary or peripheral artery disease. Eur Heart J Cardiovasc Pharmacother 6:159-166. <https://doi.org/10.1093/ehjcvp/pvz047>

24. Baker WL, Beyer-Westendorf J, Bunz TJ, Eriksson D, Meinecke AK, Sood NA, Coleman CI (2019) Effectiveness and safety of rivaroxaban and warfarin for prevention of major adverse cardiovascular or limb events in patients with non-valvular atrial fibrillation and type 2 diabetes. Diabetes Obes Metab 21:2107-2114. <https://doi.org/10.1111/dom.13787>

25. Sjalander S, Sjogren V, Renlund H, Norrving B, Sjalander A (2018) Dabigatran, rivaroxaban and apixaban vs. high TTR warfarin in atrial fibrillation. Thromb Res 167:113-118. <https://doi.org/10.1016/j.thromres.2018.05.022>

26. Briasoulis A, Inampudi C, Akintoye E, Alvarez P, Panaich S, Vaughan-Sarrazin M (2018) Safety and Efficacy of Novel Oral Anticoagulants Versus Warfarin in Medicare Beneficiaries With Atrial Fibrillation and Valvular Heart Disease. J Am Heart Assoc 7. <https://doi.org/10.1161/JAHA.118.008773>

27. Lee HF, Chan YH, Tu HT, Kuo CT, Yeh YH, Chang SH, Wu LS, See LC (2018) The effectiveness and safety of low-dose rivaroxaban in Asians with non-valvular atrial fibrillation. Int J Cardiol 261:78-83. <https://doi.org/10.1016/j.ijcard.2018.03.063>

28. Gibson CM, Mehran R, Bode C, Halperin J, Verheugt FW, Wildgoose P, Birmingham M, Ianus J, Burton P, van Eickels M, Korjian S, Daaboul Y, Lip GY, Cohen M, Husted S, Peterson ED, Fox KA (2016) Prevention of Bleeding in Patients with Atrial Fibrillation Undergoing PCI. N Engl J Med 375:2423-2434. <https://doi.org/10.1056/NEJMoa1611594>

29. Mueller T, Alvarez-Madrazo S, Robertson C, Wu O, Bennie M (2019) Comparative safety and effectiveness of direct oral anticoagulants in patients with atrial fibrillation in clinical practice in Scotland. Br J Clin Pharmacol 85:422-431. <https://doi.org/10.1111/bcp.13814>

30. Vranckx P, Valgimigli M, Eckardt L, Tijssen J, Lewalter T, Gargiulo G, Batushkin V, Campo G, Lysak Z, Vakaliuk I, Milewski K, Laeis P, Reimitz PE, Smolnik R, Zierhut W, Goette A (2019) Edoxaban-based versus vitamin K antagonist-based antithrombotic regimen after successful coronary stenting in patients with atrial fibrillation (ENTRUST-AF PCI): a randomised, open-label, phase 3b trial. Lancet 394:1335-1343. <https://doi.org/10.1016/S0140-6736(19)31872-0>

31. Oldgren J, Steg PG, Hohnloser SH, Lip GYH, Kimura T, Nordaby M, Brueckmann M, Kleine E, Ten Berg JM, Bhatt DL, Cannon CP (2019) Dabigatran dual therapy with ticagrelor or clopidogrel after percutaneous coronary intervention in atrial fibrillation patients with or without acute coronary syndrome: a subgroup analysis from the RE-DUAL PCI trial. Eur Heart J 40:1553-1562. <https://doi.org/10.1093/eurheartj/ehz059>

32. Hohnloser SH, Oldgren J, Yang S, Wallentin L, Ezekowitz M, Reilly P, Eikelboom J, Brueckmann M, Yusuf S, Connolly SJ (2012) Myocardial ischemic events in patients with atrial fibrillation treated with dabigatran or warfarin in the RE-LY (Randomized Evaluation of Long-Term Anticoagulation Therapy) trial. Circulation 125:669-676. <https://doi.org/10.1161/CIRCULATIONAHA.111.055970>

33. Bahit MC, Lopes RD, Wojdyla DM, Hohnloser SH, Alexander JH, Lewis BS, Aylward PE, Verheugt FW, Keltai M, Diaz R, Hanna M, Granger CB, Wallentin L (2013) Apixaban in patients with atrial fibrillation and prior coronary artery disease: insights from the ARISTOTLE trial. Int J Cardiol 170:215-220. <https://doi.org/10.1016/j.ijcard.2013.10.062>

34. Larsen TB, Rasmussen LH, Skjoth F, Due KM, Callreus T, Rosenzweig M, Lip GY (2013) Efficacy and safety of dabigatran etexilate and warfarin in "real-world" patients with atrial fibrillation: a prospective nationwide cohort study. J Am Coll Cardiol 61:2264-2273. <https://doi.org/10.1016/j.jacc.2013.03.020>

35. Graham DJ, Reichman ME, Wernecke M, Zhang R, Southworth MR, Levenson M, Sheu TC, Mott K, Goulding MR, Houstoun M, MaCurdy TE, Worrall C, Kelman JA (2015) Cardiovascular, bleeding, and mortality risks in elderly Medicare patients treated with dabigatran or warfarin for nonvalvular atrial fibrillation. Circulation 131:157-164. <https://doi.org/10.1161/CIRCULATIONAHA.114.012061>

36. Lauffenburger JC, Farley JF, Gehi AK, Rhoney DH, Brookhart MA, Fang G (2015) Effectiveness and safety of dabigatran and warfarin in real-world US patients with non-valvular atrial fibrillation: a retrospective cohort study. J Am Heart Assoc 4. <https://doi.org/10.1161/JAHA.115.001798>

37. Seeger JD, Bykov K, Bartels DB, Huybrechts K, Zint K, Schneeweiss S (2015) Safety and effectiveness of dabigatran and warfarin in routine care of patients with atrial fibrillation. Thromb Haemost 114:1277-1289. <https://doi.org/10.1160/TH15-06-0497>

38. Chan YH, Yen KC, See LC, Chang SH, Wu LS, Lee HF, Tu HT, Yeh YH, Kuo CT (2016) Cardiovascular, Bleeding, and Mortality Risks of Dabigatran in Asians With Nonvalvular Atrial Fibrillation. Stroke 47:441-449. <https://doi.org/10.1161/STROKEAHA.115.011476>

39. Villines TC, Schnee J, Fraeman K, Siu K, Reynolds MW, Collins J, Schwartzman E (2015) A comparison of the safety and effectiveness of dabigatran and warfarin in non-valvular atrial fibrillation patients in a large healthcare system. Thromb Haemost 114:1290-1298. <https://doi.org/10.1160/TH15-06-0453>

40. Coleman CI, Peacock WF, Antz M (2018) Comparative Effectiveness and Safety of Apixaban and Vitamin K Antagonist Therapy in Patients With Nonvalvular Atrial Fibrillation Treated in Routine German Practice. Heart Lung Circ 27:390-393. <https://doi.org/10.1016/j.hlc.2017.04.002>

41. Go AS, Singer DE, Toh S, Cheetham TC, Reichman ME, Graham DJ, Southworth MR, Zhang R, Izem R, Goulding MR, Houstoun M, Mott K, Sung SH, Gagne JJ (2017) Outcomes of Dabigatran and Warfarin for Atrial Fibrillation in Contemporary Practice: A Retrospective Cohort Study. Ann Intern Med 167:845-854. <https://doi.org/10.7326/M16-1157>

42. Lai CL, Chen HM, Liao MT, Lin TT, Chan KA (2017) Comparative Effectiveness and Safety of Dabigatran and Rivaroxaban in Atrial Fibrillation Patients. J Am Heart Assoc 6. <https://doi.org/10.1161/JAHA.116.005362>

43. Norby FL, Bengtson LGS, Lutsey PL, Chen LY, MacLehose RF, Chamberlain AM, Rapson I, Alonso A (2017) Comparative effectiveness of rivaroxaban versus warfarin or dabigatran for the treatment of patients with non-valvular atrial fibrillation. BMC Cardiovasc Disord 17:238. <https://doi.org/10.1186/s12872-017-0672-5>

44. Palamaner Subash Shantha G, Mentias A, Inampudi C, Kumar AA, Chaikriangkrai K, Bhise V, Deshmukh A, Patel N, Pancholy S, Horwitz PA, Mickelsen S, Bhave PD, Giudici M, Oral H, Vaughan Sarrazin MS (2017) Sex-Specific Associations of Oral Anticoagulant Use and Cardiovascular Outcomes in Patients With Atrial Fibrillation. J Am Heart Assoc 6. <https://doi.org/10.1161/JAHA.117.006381>

45. Burnham KT, Yang T, Wooster J (2023) A Real-World Comparison of Apixaban and Rivaroxaban in Obese and Morbidly Obese Patients With Nonvalvular Atrial Fibrillation. J Pharm Pract 37:8971900231202643. <https://doi.org/10.1177/08971900231202643>

46. Pereira MP, Lima EG, Pitta FG, Gowdak LHW, Mioto BM, Carvalho LNS, Darrieux F, Mejia OAV, Jatene FB, Serrano CV, Jr. (2023) Rivaroxaban versus warfarin in postoperative atrial fibrillation: Cost-effectiveness analysis in a single-center, randomized, and prospective trial. JTCVS Open 15:199-210. <https://doi.org/10.1016/j.xjon.2023.05.006>

47. Dari L, Beradid S, Constans J, Pariente A, Renoux C (2025) Effectiveness and safety of rivaroxaban vs. apixaban in patients with atrial fibrillation and peripheral artery disease. Eur Heart J Cardiovasc Pharmacother 11:664-673. <https://doi.org/10.1093/ehjcvp/pvaf063>

48. Kreutz R, Deray G, Floege J, Gwechenberger M, Hahn K, Luft AR, Persson P, Axthelm C, Beer JH, Bergler-Klein J, Lellouche N, Taggeselle J, Coleman CI, Beyer-Westendorf J, registry X (2024) Rivaroxaban vs Vitamin K Antagonist in Patients With Atrial Fibrillation and Advanced Chronic Kidney Disease. JACC Adv 3:100813. <https://doi.org/10.1016/j.jacadv.2023.100813>

49. Liu P (2024) A comparative study of the clinical benefits of rivaroxaban and dabigatran in patients with nonvalvular atrial fibrillation with high bleeding risk. Front Cardiovasc Med 11:1445970. <https://doi.org/10.3389/fcvm.2024.1445970>

50. Fang CW, Hsieh CY, Yang HY, Tsai CF, Sung SF (2025) Comparative effectiveness and safety of direct oral anticoagulants and warfarin in atrial fibrillation patients with dementia. Eur Stroke J 10:128-136. <https://doi.org/10.1177/23969873241274598>
